# Performance of computer scientists in the assessment of thyroid nodules using TIRADS lexicons

**DOI:** 10.1007/s40618-024-02518-9

**Published:** 2024-12-18

**Authors:** P. Trimboli, A. Colombo, E. Gamarra, L. Ruinelli, A. Leoncini

**Affiliations:** 1 Clinic for Endocrinology and Diabetology, Thyroid Unit, Ente Ospedaliero Cantonale (EOC), Bellinzona, Switzerland; 2 Faculty of Biomedical Sciences, Università Della Svizzera Italiana, Lugano, Switzerland; 3 Clinical Trial Unit, Ente Ospedaliero Cantonale (EOC), Bellinzona, Switzerland; 4 Team Innovation and Research, Area ICT, Ente Ospedaliero Cantonale (EOC), Bellinzona, Switzerland; 5 Clinic for Radiology, Imaging Institute of Southern Switzerland, Ente Ospedaliero Cantonale (EOC), Bellinzona, Switzerland

**Keywords:** Thyroid, Ultrasound, TIRADS, Artificial Intelligence

## Abstract

**Objectives:**

Ultrasound (US) evaluation is recognized as pivotal in assessing the risk of malignancy (RoM) of thyroid nodules (TNs). Recently, various US-based risk-classification systems (Thyroid Imaging and Reporting Data Systems [TIRADSs] have been developed. An important ongoing project concerns the creation of an international system (I-TIRADS) using unique terminology. Since online tool allow clinicians and patients to stratify the RoM of any TN, the role of computer scientist (CS) should be relevant. This study explored the performance of CS in assessing TNs across the TIRADS categories.

**Methods:**

The most diffused TIRADSs (i.e., ACR, EU, and K) were considered. Three-hundred scenarios were created. A CS was asked to assess the 300 TNs according to ACR-, EU-, and K-TIRADS. These data were compared with that of clinicians. The inter-observer agreement was estimated with Cohen kappa (κ). Word-cloud plots were used to graph the US descriptors with disagreement.

**Results:**

The correspondence of the CS’s assessment with the physicians was 100%, 81%, and 43%, using ACR-, EU-, and K-TIRADS, respectively. The CS was unable to classify 19/100 TNs according to EU-TIRADS and 15/100 TNs according to K-TIRADS. The inter-observer agreement between CS and physicians was excellent for ACR-TIRADS (κ = 1), moderate for EU-TIRADS (κ = 0.56), and fair for K-TIRADS (κ = 0.22). Among the non-concordant cases, 16/22 descriptors for EU-TIRADS and 18/18 descriptors for K-TIRADS were found.

**Conclusion:**

CSs are confident with the ACR-TIRADS lexicon and structure while not with EU- and K-TIRADS, probably because they are pattern-based systems requiring medical training.

## Introduction

Thyroid nodules (TNs) are a very frequent pathological condition. Since the vast majority of TNs are benign, accurate rule-out strategies to select the few cases that require further diagnostic work-up are required. Ultrasound (US) evaluation is universally recognized as pivotal in assessing the risk of malignancy (RoM) for TNs [1]. Certain US features are considered to indicate significant risk, and their specificity increases when they are used in combination. Recent years have seen the development of various US-based risk-classification systems, namely Thyroid Imaging and Reporting Data Systems (TIRADSs) [2–4]. The aims of TIRADSs are to 1) standardize the lexicon used in thyroid US reports; 2) facilitate TN risk assessment; and 3) improve the selection of cases advanced to fine-needle aspiration cytology (FNAC) assessment. As the reliability of TIRADSs has been demonstrated in the literature [5, 6], these systems have adopted rapidly worldwide. However, with this widespread diffusion, differences between TIRADSs have emerged, largely due to differences in geographical and cultural context, the combination of US features in certain categories, and the resulting category-specific RoM. Most important, however, is the difference in the terminology used to define the US characteristics between TIRADSs. Accordingly, given that all TIRADSs are reliable in clinical practice and have improved diagnostic accuracy, an important ongoing international project concerns the creation of a universal lexicon (phase I) and, finally, a universal TIRADS, namely I-TIRADS (phase II) [7]. Since phase I is pivotal, and considering that the differences in terminology used in TIRADSs are non-negligible, it is necessary to consider whether the terminologies proposed in the three major TIRADSs [2–4] are easily understandable and interchangeable for thyroid US operators. In addition, US operators and clinicians must consider how easily computer scientists (CSs) can understand US terminology, as they are often involved in the development of algorithms and automatic TN classification systems. Indeed, many online sites and applications based on TIRADSs that allow clinicians and patients to stratify the RoM of any TN have recently become available. Thus, investigating the performance of CSs in classifying TNs according to the TIRADSs is an intriguing research area in the current era.

The present study was undertaken to explore the performance of CS in assessing TNs across the TIRADS categories. Multiple cases of TNs described according to the three major TIRADSs [2–4] were created. The findings of CSs were compared to those of expert US operators, and inter-observer agreement was calculated.

## Methods

### Study design

**Fig. 1:**
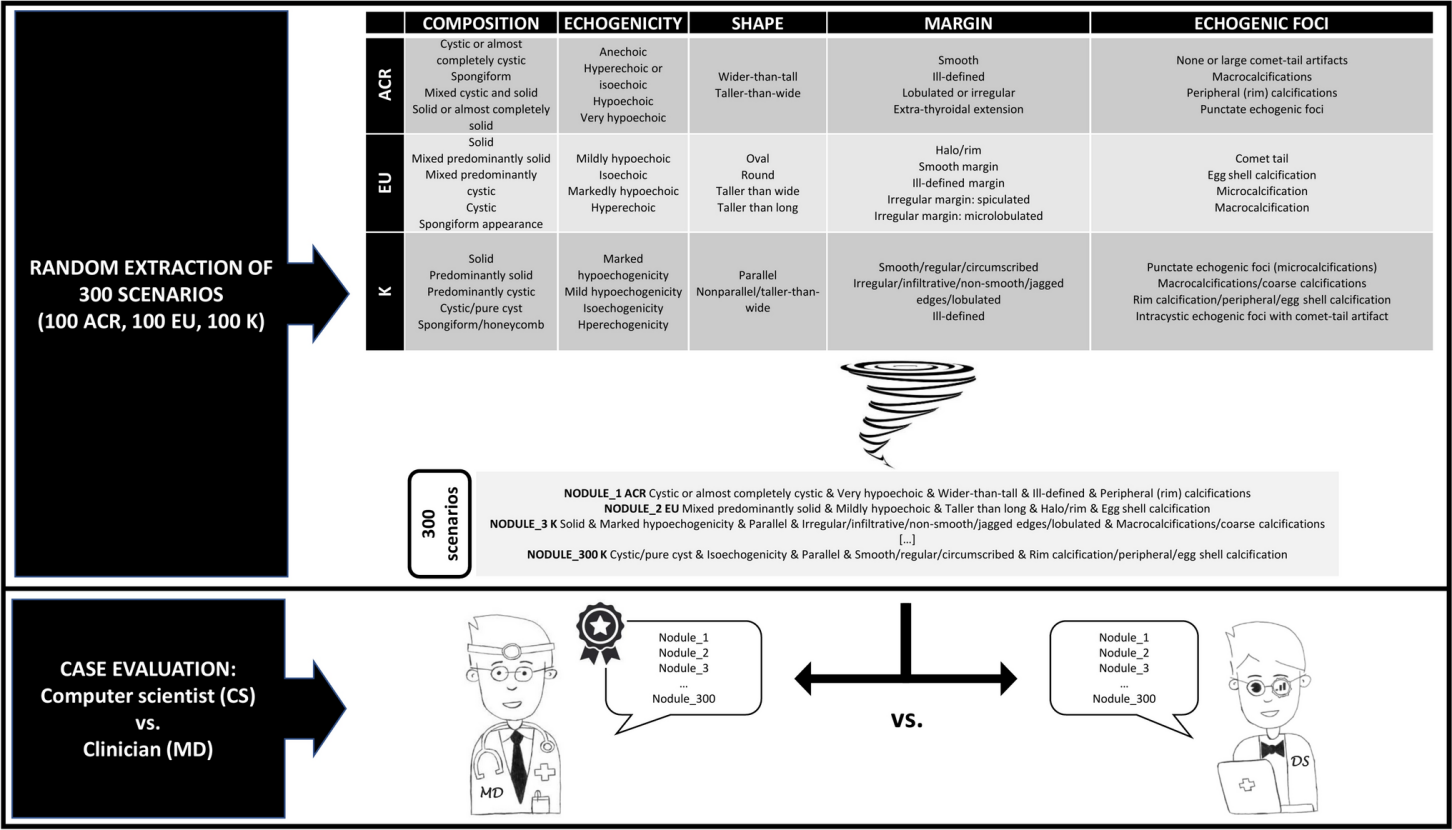
Schematic representation of the study design

The terminologies of ACR-TIRADS [2], EU-TIRADS [3], and K-TIRADS [4] were fully explored. Their categories (composition, echogenicity, margin, shape, and echogenic foci) and the associated descriptors were summarized and aligned in a single file. The study design included a five-step procedure (Fig. 1) as follows: 1) A senior CS (LR) created 100 scenarios for each of the three TIRADS randomly combining the descriptors of the categories. This phase was conducted with the aim of avoiding the generation of predefined subgroups with comparable sample sizes. 2) A second CS (AC) read the reports for the 300 cases and assessed them according to TIRADS. 3) A senior radiologist (AL) read the reports and assessed them blind of the response of the CS. 4) A senior endocrinologist (PT) reviewed the radiologist’s assessments. Cases of disagreement were discussed until mutual consensus was reached. 5) Finally, the assessments of the radiologist and the CS were compared and their inter-agreement calculated.

### Ethics

This study did not include data from humans, so approval of an ethics committee was waived.

### Statistical analysis

The inter-observer agreement was estimated in terms of Cohen kappa (κ), where κ indicates the strength of agreement (κ = 1 indicates complete agreement while κ = 0 indicates no agreement) interpreted as follows: 0–0.20: none to slight; 0.21–0.40: fair; 0.41–0.60: moderate, 0.61–0.80: substantial; 0.81–1.0: almost perfect. Statistical analyses were performed using the Sklearn software package (2024, scikit-learn developers [BSD License]). The WordCloud Python library (© 2024 Python Software Foundation) was used to graph the grade of inter-observer disagreement for the US descriptors by word-cloud plots; a wordcloud is a visual representation of word data where the bigger the word appears, the more often it is frequent.

## Results

### TIRADS assessment of cases by the radiologist and endocrinologist (reference standard)

Overall, the two physicians assessed the 300 TNs according to the three TIRADSs as follows: 56 class 5, 38 class 4, 2 class 3, and 4 class 2 for ACR-TIRADS; 76 class 5, 17 class 4, 1 class 3, 6 class 2 for EU-TIRADS; and 19 class 5, 40 class 4, 25 class 3, 16 class 2 for K-TIRADS.

### TIRADS assessment of cases by the CS

The correspondence of the CS’s assessments with those of the physicians was 100% for TNs classified according to ACR-TIRADS, 81% for cases classified with EU-TIRADS, and 43% for cases assessed across K-TIRADS categories. Table 1 illustrates the result of the TN assessment of CS and physician across the three systems. The CS was unable to classify 19/100 TNs according to EU-TIRADS and 15/100 TNs according to K-TIRADS. In the remaining 42/100 K-TIRADS cases, the CS rating disagreed with the assessment of the physicians. The inter-observer agreement between the CS and physicians was excellent for ACR-TIRADS (κ = 1), moderate for EU-TIRADS (κ = 0.56), and fair for K-TIRADS (κ = 0.22). Accordingly, while the result of ACR-TIRADS was consistent, there is potential to improve consistency in EU-TIRADS. The low level of agreement in K-TIRADS may be due to inconsistencies in interpretation or guidelines unclear with CSs.

**Table 1:** Summary of TN assessment by computer scientist and physician according to ACR-, EU-, and K-TIRADS

Data Scientist	Physician														
	ACR-TIRADS					EU-TIRADS					K-TIRADS				
	2	3	4	5	NC	2	3	4	5	NC	2	3	4	5	NC
TR2	4	0	0	0	0	6	0	0	0	0	6	6	3	2	2
TR3	0	3	0	0	0	0	0	0	0	0	5	7	3	0	0
TR4	0	0	38	0	0	0	0	1	0	0	5	11	26	6	0
TR5	0	0	0	56	0	0	0	0	74	0	0	0	0	4	0
NC	0	0	0	0	0	0	1	16	2	0	0	0	8	7	0

NC, not classified

### Analysis of non-concordant cases

**Fig. 2:**
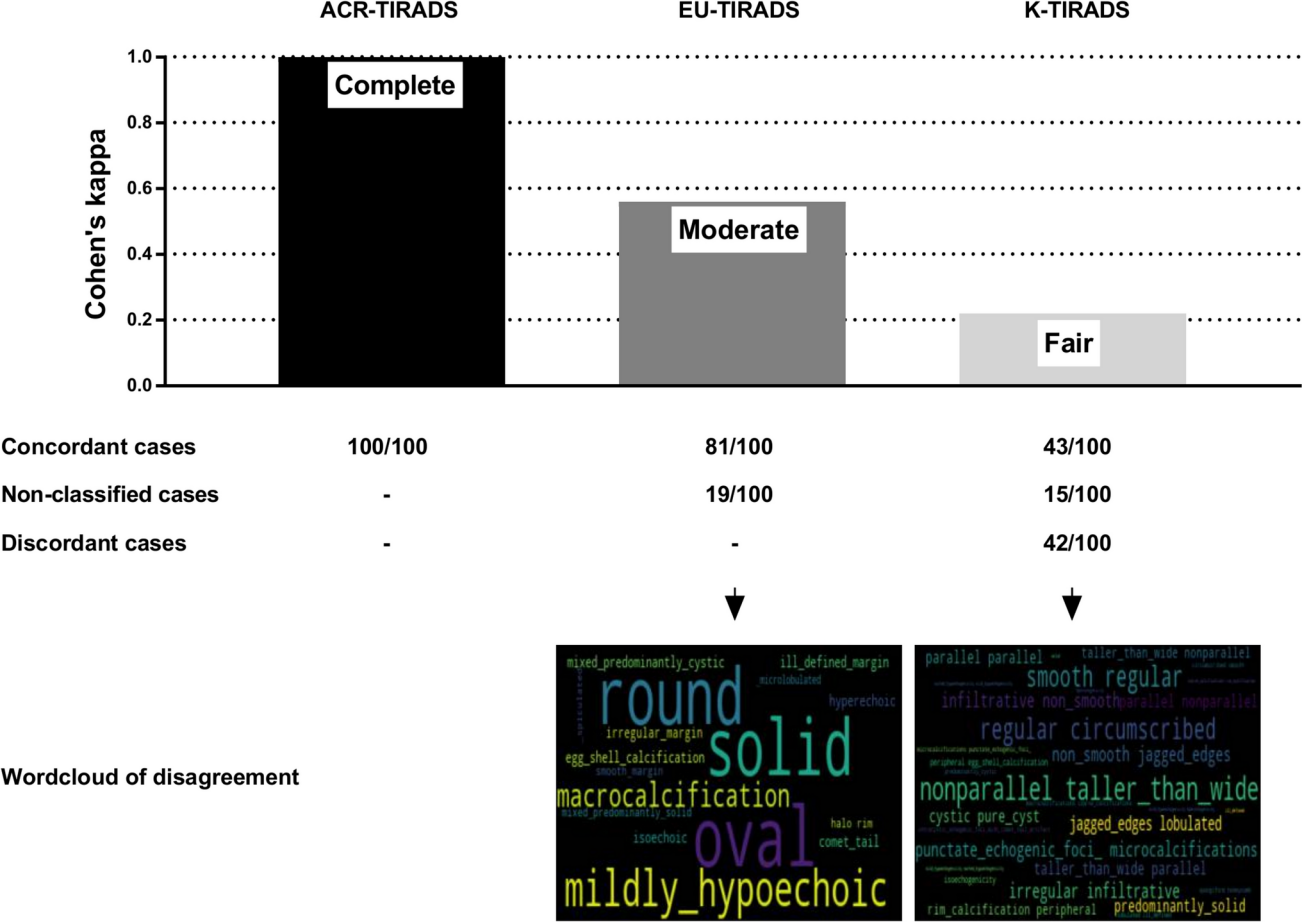
Results of the study

The non-concordant (non-classified or discordant) cases according to EU- and K-TIRADS were reviewed to analyze the frequency of terms and identify the most prevalent ones. As detailed in Table 2, among the non-concordant cases, 16/22 descriptors for EU-TIRADS and 18/18 descriptors for K-TIRADS were found. Figure 2 summarizes these results. The wordcloud plots show as bigger the most frequent terms in the nodules classified incorrectly by CS. For EU-TIRADS there were clearly predominant terms: "round" and "oval" for shape, "mildly hypoechoic" for echogenicity, and "macrocalcification" for echogenic foci. For K-TIRADS there were no clearly predominant terms causing discordance; however, terms like "taller than wide" and "nonparallel" were more prevalent, along with "regular," "smooth," and "circumscribed".

**Table 2:** Prevalence of US descriptors in non-concordant cases

EU-TIRADS (19/100 NON-CONcordant cases)		K-TIRADS (57/100 NON-CONcordant cases)	
Category: descriptor	Prevalence	Category: descriptor	Prevalence
Shape: oval	10	Shape: parallel	29 (7)
Composition: solid	9	Shape: nonparallel/taller-than-wide	28 (8)
Shape: round	9	Margin: smooth/regular/circumscribed	22 (6)
Echogenicity: mildly hypoechoic	7	Echogenic foci: punctate echogenic foci (microcalcifications)	20 (3)
Echogenic foci: egg shell calcification	7	Margin: irregular/infiltrative/non-smooth/jagged edges/lobulated	19 (4)
Composition: mixed predominantly cystic	6	Echogenicity: mild hypoechogenicity	17 (8)
Echogenicity: hyperechoic	6	Margin: ill-defined	16 (5)
Echogenicity: isoechoic	6	Echogenicity: marked hypoechogenicity	15 (7)
Margin: ill-defined margin	6	Composition: cystic/pure cyst	15 (−)
Echogenic foci: comet tail	6	Composition: predominantly solid	14 (12)
Echogenic foci: egg shell calcification	6	Echogenicity: hyperechogenicity	13 (−)
Composition: mixed predominantly solid	4	Echogenic foci: rim calcification/peripheral/egg shell calcification	13 ((4)
Margin: halo/rim	4	Echogenicity: isoechogenicity	12 (−)
Margin: smooth margin	4	Echogenic foci: intracystic echogenic foci with comet-tail artifact	12 (5)
Margin: irregular margin	3	Echogenic foci: macrocalcifications/coarse calcifications	12 (3)
Margin: irregular margin	2	Composition: spongiform/honeycomb	11 (−)
		Composition: predominantly cystic	10 (−)
		Composition: solid	7 (3)

All the 19 non-concordant cases according to EU-TIRADS were non classified. The 57 non-concordant cases according to K-TIRADS include 15 non classified cases (data in parenthesis)

## Discussion

During recent years, TIRADSs have gained worldwide acceptance because of their proven reliability and high utility in clinical practice. Thyroid and thyroid-focused international societies are making major efforts to build a unique joint TIRADS. Since current TIRADSs [2–4] include different terminologies to define the same descriptors, the goal of the International TIRADS project is to develop a generalizable lexicon [7]. Furthermore, numerous attempts at computerized analysis of both images and written texts are underway. It is therefore foreseeable that artificial intelligence (AI) may soon enter the management of thyroid US examination. Therefore, the performance of non-medical professionals, specifically CSs, is of enormous relevance for the era of AI-assisted thyroid US analysis.

The present study was undertaken to evaluate the accuracy of a CS using physicians as a gold standard. The results of this study can be summarized as follows. First, the CS performance for ACR-TIRADS was optimal, showing complete agreement with the physicians. However, significant inter-observer disagreement was found when the CS used EU- and, especially, K-TIRADS. Second, there were US scenarios that the CS could not assess according to EU- or K-TIRADS. Almost all US descriptors included in EU- and K-TIRADS were retrieved in the non-concordant cases.

To the best of our knowledge, this is the first study analyzing the performance of non-medical personnel in assessing the RoM of TNs according to US TIRADS categories and descriptors. In addition, previous studies investigating inter-observer variability between medical operators included cases with frozen US images and not their written descriptions [8–10]. Solymosi et al. evaluated the inter-observer variability between expert investigators in the judgment of ultrasound characteristics on video recording, and found a Cohen κ ranging between 0.26 and 0.71 [8]. Persichetti et al. analyzed the inter-observer variability between raters in assessing TNs according to TIRADS and found Cohen κ 0.42 for ACR-TIRADS and 0.39 for EU-TIRADS [9]. Grani et al. evaluated the inter-observer variability in indicating FNAC according to ACR-TIRADS, EU-TIRADS, and K-TIRADS and found Cohen κ 0.61, 0.68, and 0.82, respectively [10]. Here, we aimed to explore the confidence of a CS in TIRADS terminology. Since the TIRADS lexicon is a crucial issue in the development of I-TIRADS, and considering that online sites and applications have been diffused to furnish US operators (and patients) with tools to stratify the RoMs of TNs, the present findings are of interest for clinical thyroidologists and computer operators/AI specialists. Accordingly, the findings we observed merit careful discussion.

The CS’s performance was optimal when using ACR-TIRADS, which is certainly correlated with the fact that this is a point-based system. The descriptors of the categories of ACR-TIRADS are attributed a score ranging from 0 to 3; and summing the scores of the descriptors gives a final classification of the nodule across the risk categories from TR1 (benign) to TR5 (highly suspicious). This system structure facilitates risk assessment, including that by the CS, and this may be in some part due to the “mathematical” structure of the terminology.

However, the CS showed lower performance using the EU- and K-TIRADS. Both the EU- and K-TIRADS are pattern-based systems. The European system was basically conceived to discriminate high-risk cases; the presence of at least one high-risk feature classifies a TN as 5 (high risk) in EU-TIRADS. Since the US category “shape” was the most prevalent (10/19 non-concordant cases were oval and the remaining 9/19 were round), we should consider that this terminology may be confounding for non-medical figures.

The Korean system allows stratification the RoMs of TNs by a combination of descriptors. As with EU-TIRADS, the category “shape” was the most frequent between the non-concordant cases (29 parallel and 28 nonparallel/taller-than-wide).

Considering the moderate inter-observer agreement between physicians generally found in previous studies including thyroid US frozen images or clips [8–10], the present results obtained on terminology-based assessment are surprising. The agreement found between CSs and physicians in assessing TNs according to TIRADS terminology, especially ACR-TIRADS, should furnish a good base to develop further projects of AI. Basically, AI reads and learns after understanding data contained in online documents. Initially, researchers developed algorithms that imitated reasoning that humans use to solve problems. Algorithms are based on written language (informatic, clinical, popular, etc.) constantly changing according to human progress. Then, the role of humans remains essential to keep AI as much as possible autonomous in learning and developing further decisional processes. From this point of view, physicians should share their advancements with CSs and professionals involved in AI development. The present study elucidates that, currently, CSs can be aware of classification systems when the latter are conceived with a “mathematical” structure.

The prevalence of US descriptors in discordant cases between CS and physician can also be discussed on the basis of studies evaluating the inter-observer agreement between clinicians. At first glance (see Table 2), in the present study shape was the most frequent issue recorded in non-concordant cases for both EU- and K-TIRADS; and other issues such as composition, margin, and echogenic foci were often recorded in these cases. This finding seems comparable to that recorded in studies evaluating the inter-observer agreement between clinicians in assessing the single US features [11, 12, 13]. Considering that the TIRADSs were introduced just on the basis of those results with the aim to reduce the inter-observer variability between operators, the present study intrinsically confirms the need for classification systems as TIRADS. When more raters classify TNs according to their single characteristics, their variability can be higher. This should be true also for non-medical professionals depending on the terminology used to report the descriptors.

To summarize the present results, CSs can correctly interpret the lexicon of ACR-TIRADS, while this is not the case for EU- and K-TIRADS. This new data corroborates the clinical results for the three TIRADSs, which were conceived in different cultural contexts, that show different assessments of TNs [14]. In any case, it must be underlined that non-medical personnel may attempt to assess the RoMs of TNs using their written descriptions only if the descriptions are complete for all categories. Partial description of TNs including only some categories cannot supply adequate information to apply TIRADS.

The present study has potential limitations. First, the TIRADS risk classes were differently represented in the 300 cases. However, the scenarios were deliberately randomly selected to avoid the bias of predictable series. Second, the US categories for ACR-TIRADS are clearly defined as composition, echogenicity, shape, margin, and echogenic foci; while EU- and K-TIRADS do not define them as such. However, the US descriptors for the European and Korean systems allow them to be categorized comparably to ACR-TIRADS.

In conclusion, the present study shows that CSs are confident with the ACR-TIRADS lexicon and structure, and they can correctly classify TNs based on their description. This is not the case for EU- and K-TIRADS, probably because they are pattern-based systems requiring medical training.

## Data Availability

The datasets generated during and/or analyzed during the current study are not publicly available but are available from the corresponding author on reasonable request.
